# Proposal of a New Rating Concept for Digital Health Applications in Orthopedics and Traumatology

**DOI:** 10.3390/ijerph192214952

**Published:** 2022-11-13

**Authors:** Julian Scherer, Yasmin Youssef, Florian Dittrich, Urs-Vito Albrecht, Serafeim Tsitsilonis, Jochen Jung, Dominik Pförringer, Stefan Landgraeber, Sascha Beck, David A. Back

**Affiliations:** 1 Department of Traumatology, University Hospital Zurich, Raemistr. 100, 8091 Zürich, Switzerland; 2 Department of Orthopedic, Trauma and Plastic Surgery, University Hospital Leipzig, Liebigstr. 20, 04103 Leipzig, Germany; 3 Joint Centre Bergisch Land, Department for Orthopaedics, Sana Fabricius Clinic Remscheid, Brüderstraße 65, 42853 Remscheid, Germany; 4 Department of Orthopedics and Orthopedic Surgery, Universität des Saarlandes-Campus Homburg, Kirrberger Straße, 66421 Homburg, Germany; 5 Medizinische Fakultät OWL, AG 4-Digitale Medizin, Universität Bielefeld, 33501 Bielefeld, Germany; 6 Center for Musculoskeletal Surgery, Charité–Universitätsmedizin Berlin, Augustenburger Pl. 1, 13353 Berlin, Germany; 7 ATOS Klinik Heidelberg, Department of Orthopaedic Surgery, Bismarckstr. 9-15, 69115 Heidelberg, Germany; 8 Trauma Surgery, Klinikum rechts der Isar, Technical University of Munich, Clinic and Policlinic for Trauma Surgery, Ismaninger Str. 22, 81675 Munich, Germany; 9 Clinic for Orthopaedics and Trauma Surgery, Sportsclinic Hellersen, Paulmannshöher Str. 17, 58515 Lüdenscheid, Germany; 10 Bundeswehr Hospital Berlin, Department for Traumatology and Orthopedics, Scharnhorststr. 13, 10115 Berlin, Germany

**Keywords:** orthopedic society, health-related apps, mHealth, telemedicine, healthcare certification, quality standard, patient safety

## Abstract

Background: Health-related mobile applications (apps) are rapidly increasing in number. There is an urgent need for assessment tools and algorithms that allow the usability and content criteria of these applications to be objectively assessed. The aim of this work was to establish and validate a concept for orthopedic societies to rate health apps to set a quality standard for their safe use. Methods: An objective rating concept was created, consisting of nine quality criteria. A self-declaration sheet for app manufacturers was designed. Manufacturers completed the self-declaration, and the app was examined by independent internal reviewers. The pilot validation and analysis were performed on two independent health applications. An algorithm for orthopedic societies was created based on the experiences in this study flow. Results: “Sprunggelenks-App“ was approved by the reviewers with 45 (98%) fulfilled criteria and one (2%) unfulfilled criterion. “Therapie-App” was approved, with 28 (61%) met criteria, 6 (13%) unfulfilled criteria and 12 (26%) criteria that could not be assessed. The self-declaration completed by the app manufacturer is recommended, followed by a legal and technical rating performed by an external institution. When rated positive, the societies’ internal review using independent raters can be performed. In case of a positive rating, a visual certification can be granted to the manufacturer for a certain time frame. Conclusion: An objective rating algorithm is proposed for the assessment of digital health applications. This can help societies to improve the quality assessment, quality assurance and patient safety of those apps. The proposed concept must be further validated for inter-rater consistency and reliability.

## 1. Introduction

Digitization has become an integral part of everyday life due to the expansion of the capabilities of the Internet as well as the use of smartphones and corresponding mobile applications (apps) [1]. Apps can facilitate daily life on many levels and have also found their place in the healthcare sector. Consequently, patients and physicians thrive on gaining maximum advantages from digital mobile health products in terms of screening, diagnostics, therapy and rehabilitation [2,3]. The World Health Organization (WHO) has stressed that new technologies should be employed to benefit from the full potential of digitalization. The topic’s relevance was further increased by the current COVID-19 pandemic [4]. There are many “fitness” and “lifestyle” or even diagnostic and therapy-related smartphone apps available [5]. However, the offer in the major app stores is extensive and confusing; for example, in July 2022, there were 54,603 apps in the category “Medical” in the Google^®^ Play Store alone [6]. The highly liberal app markets have a drawback, as they are poorly regulated. App quality control is minimal [7]. In addition to that, the rapid development of health-related apps makes it increasingly difficult for consumers, physicians, and healthcare organizations to identify appropriate and high-quality apps in commercial app stores [8,9]. While apps are presented with a star rating system in commercial app stores, there is no indication of their professional and technical quality. Star rating systems can be an indicator of user satisfaction, which does not necessarily equate with the clinical quality and safety of the application [10]. There are also concerns about inappropriate and inaccurate health-related apps compromising or negatively effecting the user’s health and safety [11,12,13]. Several studies have, therefore, aimed to elaborate assessment criteria and tools for health-related apps [14]. However, assessment criteria also seem to be very heterogenous, which might reflect on different assessment approaches. Over the years, several players from the private and public sectors have attempted to improve the lack of quality and regulation on the app market, but none of these attempts prevailed. One exception is the regulatory scheme for medical devices that only applies to a very limited number of apps [15]. Passing the regulatory process is one possible criterion for quality, but “quality” comprises many more aspects, such as technical soundness, as proof of basic functionality. Clearly, there is a need for criteria with which users (healthy laypeople, patients or medical staff) can objectively identify whether certain app products are suitable for their own needs [16,17].

In some countries, it has been proposed to create prescription-based smartphone apps that medical insurance can cover. Worldwide, Germany was the first country to recommend introducing the prescription of digital health applications via federal law. This increased the need for adequate quality assessments [18,19,20]. Here, the certification of medical products is well established and has proven to contribute to quality enhancement in various fields of the healthcare system [21,22].

There are different approaches to creating an objective evaluation of apps based on defined quality criteria [7]. Some institutions are already pursuing the path of certification based on predefined quality criteria, such as the NHS App library or the Diabetes App seal “DiaDigital” of the German Diabetes Associations [23,24,25]. While validated test procedures are available [26,27,28], literature research on PubMed^®^ using the search terms “certificate + mobile app” only revealed 77 results (as of October 2022) with very heterogenic approaches.

An appropriate solution for quality assurance of medical and health-related apps that equally considers the character of the market, the technology, the use, the users, and the setting is still missing but needed [11]. A unified set of criteria for the self-declaration of the quality of health apps was created in 2019 [29].

Previous data have shown that smartphone apps are still underused in the medical sector but could enhance patient satisfaction and communication between patients and physicians, as well as the quality of care [30]. In orthopedics, studies have revealed that patients would be willing to use telehealth and smartphone applications [31,32]. Furthermore, smartphone apps are used, e.g., to support the process of lower-back pain treatment and proved not to be inferior to conventional physiotherapy [33]. However, these apps may lack medical evidence and professional involvement, which results in the demand for the regulation and evaluation of such apps [34].

Thus, this work aimed to establish and validate a user-friendly rating tool for health-related apps in the field of orthopedics, which can be used by orthopedic societies to certify apps, thereby setting quality standards in terms of clinical quality and safe usage of apps of both patients and physicians.

## 2. Materials and Methods

### 2.1. Experimental Setting

To create a workable, stable, validated and clearly structured concept for app rating, a review of the previously published literature was conducted on PubMed^®^. 

In accordance with AWMF recommendations (Arbeitsgemeinschaft der Wissenschaftlichen Medizinischen Fachgesellschaften e.V. or Association of the Scientific Medical Societies in Germany), the proposed concept is based on nine central quality principles, including applicable sub-principles for the rating of medical applications [29]. Besides the main criteria, sub-criteria of each main criteria were developed (e.g., (A) “risk awareness” and (B) “risk handling” in “Appropriateness of Risk”). This chosen app rating concept has been evaluated in several publications. The central principles are presented and elucidated in Table 1 [15,35].

Since there are no examples in the digital or print literature, a self-declaration sheet was created based on these principles that must be completed by the application manufacturer.

To ease the process of app rating for orthopedic societies and to make the procedures transparent for manufacturers, an evaluation sheet was created, which must be filled in by appointed representatives of the respective orthopedic/traumatological society to evaluate the application.

### 2.2. Pilot Validation

For the validation process we used a sample of two apps. For the analysis, we approached two independent health-application manufacturers:

1. Sprunggelenks-App (“Ankle-Joint-App”), Mediploy GmBH, 40764 Langenfeld, Germany.
2. Therapie-App (“Therapy app”), Bauerfeind^®^, 07937 Zeulenroda-Triebes, Germany.

Both manufacturers were informed that the evaluation process was only for study purposes and that no certification would be granted in case of positive validation.

**Figure 1:**
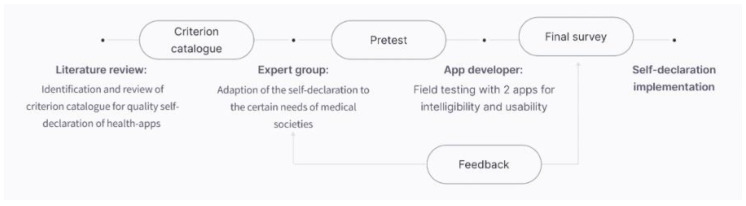
Workflow of the study.

After receiving the self-declaration reports of the manufacturers, each application was rated by the coauthors S.T. and S.B. for validation [36]. The workflow of the study is depicted in Figure 1.

### 2.3. Recommendations for Orthopedic Societies

Based on the experiences and findings of the chosen approach, a strategy for a potential procedure for orthopedic societies was created on how to create an internal validation and recommendation process for patients and colleagues about relevant apps in their field.

### 2.4. Justification of Methods Used

The synthesis of the structured concept for app rating was based on well-established quality criteria, which makes our concept robust and pre-validated. A pilot validation was performed using two independent app manufactures who were naïve to the proposed self-declaration sheet, which eradicated any bias.

## 3. Results

The free PubMed search revealed 1256 published articles in 2020. Most of the studies focused on the assessment of the respective health application by patients. No publication described a standardized certification algorithm for medical apps.

### 3.1. Self-Declaration Sheet

A self-declaration sheet (see Table 2) for the manufacturer was synthesized based on the previously published criteria for the self-declaration of the quality of health apps [29]. The self-declaration sheet is based on the previously published nine quality criteria. Sub-criteria were restructured according to the need to rate digital health applications in the fields of orthopedics and traumatology. Each quality principle is explained briefly to make the self-declaration sheet better understandable (Table 2).

### 3.2. Society Evaluation Sheet

The society evaluation sheet (see Table 3) focuses on the primary data of the proposed health app (manufacturer details, the purpose of the proposed application, technical evaluation, etc.) Furthermore, every sub-criterion within the quality principles can be rated as fulfilled (green), not fulfilled (red) or not assessable (yellow). In addition, six sub-criteria regarding the test quality criteria must be answered accordingly. At the end of the sheet, the reviewer can add general recommendations and further comments and classify the proposed app as approved or not approved.

### 3.3. Pilot Validation

Besides the app’s “demographics” (store availability, aim, specifications, etc.), there were 46 sub-criteria to be answered regarding the manufacturer’s fulfilment. Since this was a pilot validation, the technical assessment by an external company or society was not performed. “Sprunggelenks-App“ (*Ankle app*) by Mediploy GmbH was approved by the reviewer and was rated with 45 (98%) fulfilled criteria and one (2%) not fulfilled criterion. The reviewers also approved “Therapie-App” (Therapy app) by Bauernfeind^®^. However, the application was rated with 28 (61%) criteria that were met, while 6 (13%) criteria were not fulfilled. An additional 12 (26%) criteria could not be assessed (Table 4). Due to data security reasons, we did not include the detailed answer sheets of the manufacturers.

### 3.4. Proposal for Society Assessment

Based on the experiences of the validation process, the below procedure is suggested.

**Figure 2:**
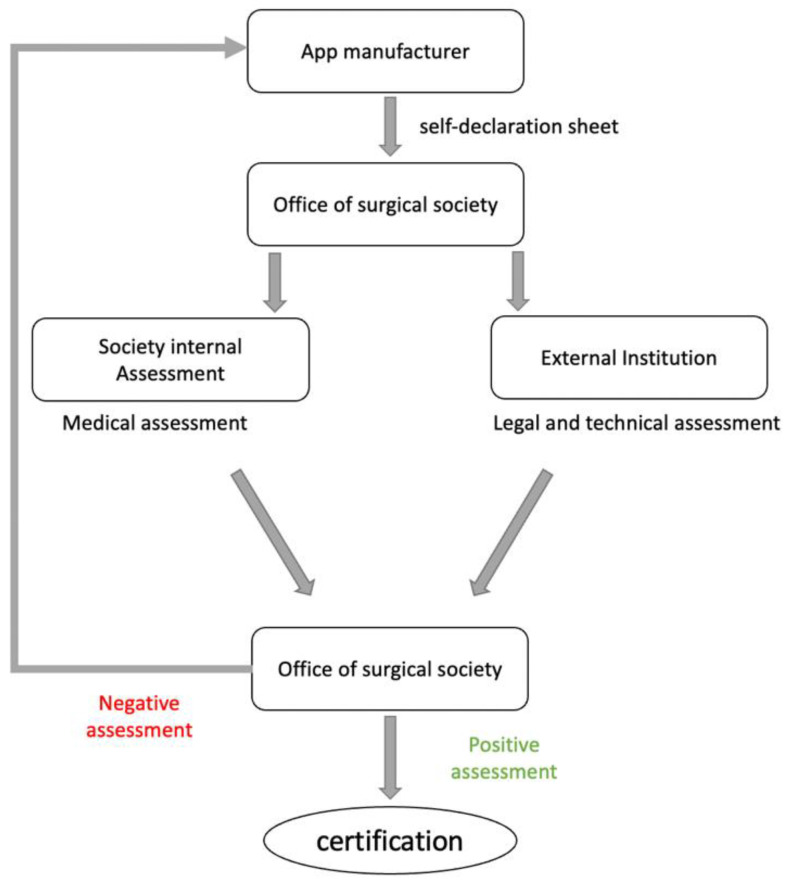
Flowchart depicting the proposed algorithm for the rating of mobile apps performed by orthopedic societies.

The evaluation process is started after the manufacturer has submitted the self-declaration form, which can be downloaded from the society’s website. If all documents are completed, the submitted application should be sent to an external institution that evaluates legal and technical aspects. If sufficient, the societies’ internal review (Table 3), using independent raters, should be performed. Independent raters for potential health apps should be chosen for the different orthopedic sections (e.g., “upper extremity” or “spine” when applicable). If rated positive, the society’s logo could be granted to the manufacturer as visual certification. If rated negative, the manufacturer should be given a period of 3 months to improve the app and/or declaration. The certificate should be valid for a limited timespan only, for example, for three years, after which a re-evaluation is required (Figure 2).

### 3.5. Practical Implications

The practical experiences of the rating process are as follows:

1. The initial reviewing process takes 2–3 h.
2. In case of uncertainties and the need to contact the manufacturer, the initial reviewing process can be significantly prolonged.
3. A coordinator between the society and the reviewer should be chosen for an initial briefing regarding the process.
4. An external institution must review the legal and technical requirements of the apps. Apparent violations of legal and technical requirements would, therefore, already lead to the exclusion of the app from the rating process, even before the professional content assessment.
5. Financial compensation for coordinators and reviewers should be considered to maintain a general willingness to review proposed health applications. We suggest that the individual manufacturer covers this.

## 4. Discussion

Using smartphones and corresponding mobile applications (apps) provides access to information and interventions at any time and in various settings. Previous studies have shown that health-related apps have the potential to make a significant difference in the health outcomes of patients [37]. However, there are also concerns about inappropriate apps that could potentially limit or even negatively affect patient health outcomes. Further concerns include privacy and data security issues [11,38,39].

The rapid development of health-related apps makes it increasingly difficult for consumers, physicians and healthcare organizations to identify appropriate and high-quality apps in commercial online application stores [8,9]. For the selection of apps, users often rely on star rating systems and user reviews provided by the respective app stores, even if these evaluation methods can be misleading [10,40]. Therefore, there is an urgent need to support consumers, both patients and recommending physicians, in identifying appropriate and safe apps.

There have been various approaches to assess health-related mobile applications in the past [41]. A common tool that has been validated is the Mobile App Rating Scale (MARS), developed as a multidimensional measure tool to pilot, classify and assess the quality of mobile health apps [27]. The MARS consists of 23 items in five subscales (engagement, functionality, aesthetics, and information quality and subjective quality). The subjective quality subscale is, however, excluded from the overall mean app quality score in this scoring system [27]. A separate version of the MARS for end users, the uMARS, was also created as a simple tool for users, to assess the quality of health-related apps [42]. On the other hand, the AWMF recommendation, which has been used for the app-rating in this study, proposes nine central quality principles for the rating of medical apps, with associated sub-criteria [29]. This allows an even more differentiated and discriminate rating of the apps to be performed, and at the same time, has fewer main objectives, which makes the proposed rating system handier. Furthermore, the MARS has been validated as a tool for the comparison of health applications rather than for the rating of specific health applications performed by, e.g., medical societies.

In this study, we introduce an algorithm for orthopedic/traumatological societies that would allow them to assess and certify the structural and content-related quality of commercial apps that focus on orthopedic/traumatological conditions. The proposed rating system is based on standardized objective items. This visual certification could help users, consumers and medical professionals to choose the appropriate apps for their needs. To the best of the authors’ knowledge, this is the first study that describes the development of an algorithm that aims to certify clinically relevant and safe apps in the field of orthopedics/ traumatology.

The award of a certificate or seal of approval goes hand in hand with an excessive level of responsibility, as users trust its validity [43]. Valid test processes usually require a strict methodical approach and the implementation of state-of-the-art test processes. Quality losses in the test are inevitable if this cannot be guaranteed in the long term. However, it should also be considered that the affixing of quality seals to medical products may be problematic in a legal sense, as at least in Europe, the CE marking does not tolerate any other markings that could potentially lead to confusion [44]. To abstain from any evaluation completely, however, contradicts the obvious need for qualitative guidance for patients and doctors from the authors’ point of view.

An app-rating process based on self-declarations is proposed. Manufacturers would have the possibility to let their applications be certified by the respective orthopedic/traumatological society. After an initial assessment of legal and technical aspects performed by an external company, the application is evaluated on content quality by a reviewer from the respective society. Following this proposed process, it could be assured that the health application is checked for correctness in terms of medical standards and quality requirements.

The legal and formal requirements are primarily in a regulatory gray area and are regularly overtaken by rapidly and dynamically developing technical innovations. A potentially faulty test identified by the user can immediately lead to a loss of integrity on the part of the testing / certifying institution.

We propose the logo “APProved by (name of the respective society)” for the visual certification of the application in case of a positive rating, albeit with a limited validity of this certification (e.g., three years). This restriction would ensure that health-related applications evaluated following this process are repeatedly checked for quality. Implications of this process are that it requires good coordination, is time intensive, and can take up to 6 h when there are uncertainties on the reviewer’s side.

A self-declaration process is proposed, as the comprehensive search for suitable health applications in the different app stores would be too strenuous for the respective societies, even when using search engines or specially programmed algorithms. Furthermore, orthopedic/traumatological societies do not have the capacity to evaluate more variables of proposed applications than the medical/technical facets (e.g., legal concerns, technical requirements). One significant advantage of this concept of self-declaration is that the technical checks of the proposed apps are performed by external institutions, which reduces the workload for the orthopedic/traumatological society, which would only be responsible for strict medical feasibilities. Furthermore, the respective society cannot be made liable for legal inconsistencies.

However, there were also limitations to this study. First, only two health-related apps were rated in this study by only two reviewers for pilot validation. The consistency and inter-rater reliability could, therefore, not be evaluated. Furthermore, only one certifying approach was examined and tested. Alternative frameworks for certification processes, including other rating scales, were not considered.

## 5. Conclusions 

Due to an increasing number of medical smartphone applications (apps), there is an urgent need for an objective quality assessment for both usability and content. The proposed rating algorithm for digital health applications in orthopedics and traumatology can help societies to improve the quality assessment and quality assurance of those apps. The proposed concept should be further tested for inter-rater consistency and reliability and should be validated in future studies.

## Figures and Tables

**Table 1:** Detailed explanation of the nine central quality principles for medical apps, as proposed by AWMF.

No.	Quality Principle	Explanation
**I**	**Transparency**	Valid, reliable and target-group-specific information is available on the quality requirements that the health app and underlying software meet to support their evaluation as well as individual and collective usage decisions.
**II**	**Practicality**	The health app is suitable for the intended purpose and opens corresponding areas of application and context. Proof of fulfilling the stated purpose, i.e., the benefit, is provided using appropriate methods. It is also clearly recognizable for which purpose and user group the health app is unsuitable.
**III**	**Risk adequacy**	The health app can be used without exposing the user or his environment to disproportionate health, social or economic risk. Appropriate methods are chosen to demonstrate the relationship between the benefits and the risks. The manufacturer takes precautions to rule out risks of use as far as possible.
**IV**	**Ethical soundness**	The health app is ethically sound regarding development, distribution, operation and use (e.g., concerning professional and/or research ethics). Discrimination and stigmatization of users are avoided.
**V**	**Legal conformity**	The health app is legally compliant (including medical device law, professional law and data protection law). Legal compliance is guaranteed with respect to development, distribution, operation and use.
**VI**	**Content validity**	The health-related content used and presented in the health app is up to date, valid and trustworthy. The manufacturer ensures that the content is regularly updated in a manner that is recognizable to the user and is up to date with the latest knowledge and regulatory requirements.
**VII**	**Technical adequacy**	The health app is state of the art regarding development, operation, maintenance and use and guarantees sustainability in the sense of maintainability, portability, interoperability and compatibility.
**VIII**	**Usability**	The health app enables target-group-specific use (e.g., in terms of accessibility and individualization). In addition, there is the possibility of contacting the manufacturer and/or other affected persons or health professionals if anything is unclear.
**IX**	**Resource efficiency**	The health app is resource efficient to use (e.g., in terms of power consumption, computing power, memory, data transfer, time and costs).

**Table 2:** Self-declaration sheet for app manufacturers. Application for acceptance of the app review.

No.	Quality Principle	Basic Requirements for Self-Declaration
**1**	**Transparency**	The self-declaration must provide information about the app in accordance with the “transparency” quality principle to be sufficient as a basis for individual and collective usage decisions and evaluations of the software.
	(A) Completeness of the information	
**No.**	**Questions and manufacturer information**	
**A1**	Name of the application	
**A2**	Version of the application and date of upload/update	
**A3**	Costs for the user	
**A4**	On which operational systems can the app be used? (URLs)	
**A5**	Provide manufacturer’s name and contact details	
**A6**	Describe the financing model (e.g., adverts, in-app purchases)	
**2**	**Expediency**	The self-declaration for the quality principle “Expediency” should show the extent to which the software is appropriate for the intended purpose.
	(A) Intended use	
	(B) Purpose fulfillment (C) Evidence or information on the fulfillment of the purpose / suitability	
**No.**	**Questions and manufacturer information**	
**A1**	Give a short description of your app (approx. 100 words) that clearly shows the purpose and the goals (target groups of your app, limitations or limits of the app use?):	
**B1**	With which technical functions or content-related methods contained in your app (e.g., implementation of scores, training plans, etc.) do you ensure that the goal of your app is achieved?	
**B2**	Does your app process data or user information? If so, please specify these functions:	
**C1**	Can you provide data to prove that your app achieves the purpose to be fulfilled? If so, please provide specific sources:	
**3**	**Appropriateness of Risk**	The self-declaration for the quality principle “risk appropriateness” should show the extent to which the app can be used in a risk-appropriate manner without exposing the user or his environment to a disproportionate health, social or economic risk.
	(A) Risk awareness (B) Risk management	
**No.**	**Questions and manufacturer information**	
**A1**	Do you see potential or actual economic, social or health risks when using your app?	
**A2**	Is there a risk or the danger of a third party being endangered by using the app?	
**B1**	What measures have you taken to exclude or minimize risks and dangers that (could) arise from using the app?	
**B2**	Have you set up a risk management system (in case problems / risks for users should arise)? If so, which?	
**4**	**Ethical Clearance**	The self-declaration on the quality principle “ethical harmlessness” should show the extent to which development, supply, operation and use are ethically harmless in order to prevent discrimination and stigmatization and to enable fair access.
	(A) Compliance with ethical principles (B) Disclosure of Conflicts of Interest (C) Good scientific practice	
**No.**	**Questions and manufacturer information**	
**A1**	Before the download (homepage / store description), will the end users of your app receive clear, understandable information on purpose description, data processing (data protection provisions), conditions of use (EULA) and financing?	
**A2**	Can every end user use the app equally? What are the usage hurdles (e.g., technical understanding required)?	
**B1**	Is advertising used in your app? Is this also clearly recognizable as advertising for the end user?	
**B2**	Is there a conflict of interest (e.g., has the sponsorship influenced the content of the app, etc.)?	
**C1**	Is your app being used in a scientific context? If so, does it comply with the principles of good scientific practice and is there a valid vote from the responsible ethics committee?	
**5**	**Legal Conformity**	The self-declaration for the quality principle “legal conformity” should show the extent to which legal conformity (including medical device law, professional law, data protection law) of development, offer, operation and use is guaranteed to protect all parties involved (e.g., providers, store operators, users).
	(A) General (e.g., data protection) (B) Health related (e.g., medical device law)	
**No.**	**Questions and manufacturer information**	
**A1**	Is your app GDPR (General Data Protection Regulation) compliant? Do end users have to actively consent to the collection of personal data and are they named?	
**A2**	For what purposes are the collected data used? Do you process the data and, if necessary, pass on collected user-associated data to third parties? Are users informed about this?	
**A3**	Where is the geographic location of the server on which the app or the collected data is stored? Are the data on the server protected from unauthorized access by third parties?	
**B1**	If your app falls under the Medical Devices Act (if applicable), please indicate the associated class (justification):	
**B2**	Does your app have the current CE certification, and does it meet all of the relevant regulations for its class?	
**6**	**Content Validity**	The self-declaration for the quality principle “content validity” should show the extent to which the health-related content presented and used is valid and trustworthy.
	(A) Valid sources (B) Topicality of the content	
**No.**	**Questions and manufacturer information**	
**A1**	What sources is the content of your app based on (scientific findings, guidelines, studies)? Are these sources available to the end user?	
**B1**	How do you technically ensure that the content of your app remains up to date (e.g., when guidelines are updated)?	
**B2**	Who is responsible for updating the content and, if necessary, checking guidelines or specialist literature after changes?	
**7**	**Technical Adequacy**	The self-declaration for the quality principle “Technical adequacy” should show the extent to which development, operation, maintenance, and use correspond to the state of the art to ensure sustainability in terms of maintainability, interoperability and compatibility.
	(A) Technical timeliness (B) Platform-independent use, scalability	
**No.**	**Questions and manufacturer information**	
**A1**	How often is your app updated (when was the last update? When are you planning the next?)?	
**A2**	Do you use a secure and encrypted connection according to the latest technical standards for data transmission?	
**A3**	How do you ensure that your app remains technically up to date (e.g., when updating operating systems)? Who is responsible?	
**B1**	Is it possible for the user to transfer the data collected via the app and is this necessary (e.g., when changing the mobile platform or the device used)?	
**B2**	Does your app interact / communicate with hardware, medical device, homepage, or backend? If yes how?	
**8**	**Usability**	The self-declaration for the quality principle “Usability” is intended to show the extent to which the software enables use according to the target group and contributes to the satisfaction of the user.
	(A) Proof of usability (B) User involvement	
**No.**	**Questions and manufacturer information**	
**A1**	Can you provide evidence of the usability of your app? Has the app been tested on the target group?	
**A2**	Can your app be adapted to the needs of a target group (customization, e.g., doctors / patients with regard to content, or patients with certain diseases, magnifying glass function for older people)?	
**B1**	Have you implemented the option of a feedback function or an error reporting system via your app?	
**B2**	Are users involved in the app (e.g., the possibility of comparing them with other users, via social media, saving own results for motivation, etc.)?	
**9**	**Resource Efficiency**	The self-declaration for the “Resource Efficiency” quality principle should show the extent to which the app takes resource-efficient use into account.
	(A) Efficient use of resources	
**No.**	**Questions and manufacturer information**	
**A1**	Is a permanent internet connection necessary for the stable usability of your app after the download?	
**A2**	How much storage capacity does your app take up on the mobile device?	
**A3**	How much time does the adequate use of the app take (e.g., per day)?	

**Table 3:** Society evaluation sheet: rating sheet for internal assessment through independent internal reviewers.

Reviewer Rating Sheet:			
Title of the app to be rated: Manufacturer: Costs: Target group: Physician | Patient | Physician and Patient Category: Education/Information | Therapy | Prevention | Research | Other Purpose description (max. 100 words): The self-declaration has been completed in full: Yes / No The manufacturer ensures compliance with the quality criteria in all points: Yes / No Technical evaluation:			
**P**	**MA**	**Criteria**	**Fulfilled?**
**Transparency:**			
1	A2	Was the update / upload date within the last 2 years?	**[yes] [no] [do not know]**
	A4	App available in Applestore and Google Play Store?	**[yes] [no] [do not know]**
		**Questions answered adequately, an assessment is possible.**	**[yes] [no] [do not know]**
		**Is the content of the criterion met?**	**[yes] [no] [do not know]**
**Expediency:**			
2	A1/2	Are the purpose and goals of the app clear?	**[yes] [no] [do not know]**
	B1	Do the technical functions that the app uses to fulfill its purpose appear plausible?	**[yes] [no] [do not know]**
	B2	Do the explanations regarding the data processing appear plausible?	**[yes] [no] [do not know]**
	C1	Is suitable evidence provided that supports the information on the purpose of the app (e.g., references to studies, guidelines, tests, quality seals)?	**[yes] [no] [do not know]**
	C1/2	Is any danger for the patient through limitations and exclusion criteria securely excluded?	**[yes] [no] [do not know]**
		**Questions answered adequately, an assessment is possible.**	**[yes] [no] [do not know]**
		**Is the content of the criterion met?**	**[yes] [no] [do not know]**
**Appropriateness of Risk:**			
3	A1/2	Are relevant potential or actual risks (health, economic, social) for the users or their environment when using the app described?	**[yes] [no] [do not know]**
	B1/2	Have adequate precautions been implemented to avoid health, economic and / or social risks when using the app?	**[yes] [no] [do not know]**
		**Questions answered adequately, an assessment is possible.**	**[yes] [no] [do not know]**
		**Is the content of the criterion met?**	**[yes] [no] [do not know]**
**Ethical Clearance:**			
4	A1/2	Is the end user adequately and transparently informed about the app before downloading the app and can they use the app equally?	**[yes] [no] [do not know]**
	B1/2	Are there no relevant conflicts of interest (e.g., authors with company participation) or only those that are explained transparently and do not compromise the quality of the app?	**[yes] [no] [do not know]**
	C1	Is it described whether the app follows the principles of good scientific practice when used in a research context?	**[yes] [no] [do not know]**
		**Questions answered adequately, an assessment is possible.**	**[yes] [no] [do not know]**
		**Is the content of the criterion met?**	**[yes] [no] [do not know]**
**Legal Conformity:**			
5	A1	Is it described which relevant general legal requirements, such as requirements of data protection law, GDPR, telemedia law or commercial law are considered by the manufacturer / provider of the app and are these complied with?	**[yes] [no] [do not know]**
	A2/3	Are the purposes of the data collection plausible and are they based on a secure concept?	**[yes] [no] [do not know]**
	B1/2	If the app falls under the MDA, are all regulations met?	**[yes] [no] [do not know]**
		**Questions answered adequately, an assessment is possible.**	**[yes] [no] [do not know]**
		**Is the content of the criterion met?**	**[yes] [no] [do not know]**
**Content Validity:**			
6	A1–3	Is a plausible concept drawn up as to how the quality of the content is guaranteed (e.g., inclusion of experts in the field) and which valid sources are used (e.g., consideration of current scientific findings, guidelines)?	**[yes] [no] [do not know]**
		**Questions answered adequately, an assessment is possible.**	**[yes] [no] [do not know]**
		**Is the content of the criterion met?**	**[yes] [no] [do not know]**
**Technical Adequacy:**			
7	A1–3	Does the app correspond to the current state of art?	**[yes] [no] [do not know]**
	B1	Is there a concept to what extent it is possible to switch to a different operating system or device without losing data?	**[yes] [no] [do not know]**
		**Questions answered adequately, an assessment is possible.**	**[yes] [no] [do not know]**
		**Is the content of the criterion met?**	**[yes] [no] [do not know]**
**Usability:**			
8	A1	Can valid proof of usability or target group conformity (e.g., usability tests) be made?	**[yes] [no] [do not know]**
	A2	Can the function of the app be adapted to specific target groups / barrier-free?	**[yes] [no] [do not know]**
	B1	Can the end user interact or give feedback via the app (e.g., social media, etc.)?	**[yes] [no] [do not know]**
		**Questions answered adequately, an assessment is possible.**	**[yes] [no] [do not know]**
		**Is the content of the criterion met?**	**[yes] [no] [do not know]**
**Resource Efficiency:**			
9	A1–3	Would you describe the use of the available technical resources (e.g., required memory, computing power, internal or external sensors, power consumption, etc.) as efficient?	**[yes] [no] [do not know]**
		**Questions answered adequately, an assessment is possible.**	**[yes] [no] [do not know]**
		**Is the content of the criterion met?**	**[yes] [no] [do not know]**
**Test quality criteria of the manufacturer’s information:**			
		**Is the information about the app sufficient, i.e., is it adequately given in terms of scope and depth of information?**	**[yes] [no] [do not know]**
		**Is the content of the criterion met?**	**[yes] [no] [do not know]**
		**Is valid, i.e., complete and reliable, information given about the app?**	**[yes] [no] [do not know]**
		**Is the content of the criterion met?**	**[yes] [no] [do not know]**
		**Is the information about the app described in a way that is appropriate for the target group?**	**[yes] [no] [do not know]**
		**Is the content of the criterion met?**	**[yes] [no] [do not know]**
Overall evaluation / additional comments: Recommendation for approval: [yes] [no]			

**Table 4:** Results of the pilot validation using “Therapie-App” and “Sprunggelenks-App”. Sub-criteria were rated as fulfilled (green), not fulfilled (red) or not assessable (black).

**Title**	Sprunggelenks-App	Therapie-App
**Manufacturer**	Mediploy GmbH	Bauerfeind^®^
**Costs**	No costs	No costs
**Target group**	Physicians and patients	Patients
**Category**	Education, Information, Therapy, Prevention	Education/Information, Therapy
**Purpose description**	The purpose of this app is to optimize aftercare of patients with ankle sprains (without bone lesion) using information/education, videos, diary as well as the Cumberland Ankle Instability Tool.	Supportive information and training program for patient who received a Bauerfeind^®^ product
**Self-declaration completion**	Yes	Yes
**Compliance with quality criteria**	Yes	Yes/No
**Technical evaluation**		
**Transparency**		
A2	Yes	Yes
A4	Yes	Yes
**Are questions answered adequately, and is an assessment possible?**	Yes	Yes
**Is the content of the criterion met?**	Yes	Yes
**Expediency**		
A1/2	Yes	N/A (Target is not well defined)
B1	Yes	Yes
B2	Yes	Yes
C1	Yes	N/A (No evidence displayed in the App)
C1/2	Yes	N/A (No limitations displayed)
**Are questions answered adequately, and is an assessment possible?**	Yes	Yes
**Is the content of the criterion met?**	Yes	N/A
**Appropriateness of Risk:**		
A1/2	Yes	N/A (not displayed)
B1/2	Yes	N/A (not displayed)
**Are questions answered adequately, and is an assessment possible?**	Yes	Yes
**Is the content of the criterion met?**	Yes	N/A
**Ethical Clearance**		
A1/2	Yes	No
B1/2	Yes	No (App description suggests that the app can only be used in combination with a Bauerfeind^®^ product, but the app itself is a general training program)
C1	Yes	No
**Are questions answered adequately, and is an assessment possible?**	Yes	No
**Is the content of the criterion met?**	Yes	No
**Legal Conformity**		
A1	Yes	Yes
A2/3	Yes	Yes
B1/2	Yes	Yes
**Are questions answered adequately, and is an assessment possible?**	Yes	Yes
**Is the content of the criterion met?**	Yes	Yes
**Content Validity**		
A1–3	Yes	Yes
**Are questions answered adequately, and is an assessment possible?**	Yes	Yes
**Is the content of the criterion met?**	Yes	Yes
**Technical Adequacy**		
A1–3	Yes	Yes
B1	**no**	Yes
**Are questions answered adequately, and is an assessment possible?**	Yes	Yes
**Is the content of the criterion met?**	Yes	Yes
**Usability**		
A1	Yes	Yes
A2	Yes	**No**
B1	Yes	**No**
**Are questions answered adequately, and is an assessment possible?**	Yes	N/A
**Is the content of the criterion met?**	Yes	N/A
**Resource Efficiency**		
A1–3	Yes	N/A
**Are questions answered adequately, and is an assessment possible?**	Yes	N/A
**Is the content of the criterion met?**	Yes	N/A
**Test quality criteria:**		
Is the information about the app sufficient?	Yes	Yes (but no references within the app)
Is the content of the criterion met?	Yes	Yes
Is valid information given about the app?	Yes	Yes (but in-store description not precise)
Is the content of the criterion met?	Yes	Yes
Is the information about the app appropriate for the target group?	Yes	Yes
Is the content of the criterion met?	Yes	Yes
**Overall evaluation / additional comments**:	Overall positive, relevant topic, clear goal definition, technical criteria met, no risk for user	Nice application for therapy of musculoskeletal disorders. Innovative professional approach. Things which should be addressed in the future: - Defining the target - Research references - App-store description: App can also be used without Bauerfeind^®^ product
**Recommendation for approval:**	Yes	Yes

## Data Availability

Data are available upon reasonable request.
